# Colocalization of senescent biomarkers in deep, superficial, and ovarian endometriotic lesions: a pilot study

**DOI:** 10.1038/s41598-022-21431-w

**Published:** 2022-10-14

**Authors:** Laura Palmieri, Helena Malvezzi, Bruna Cestari, Sergio Podgaec

**Affiliations:** Hospital Israelita Albert Einstein, Instituto Israelita de Ensino e Pesquisa Albert Einstein, São Paulo, São Paulo Brazil

**Keywords:** Immunological techniques, Biological techniques, Cell biology, Health care, Medical research

## Abstract

Endometriosis is a prevalent gynecological condition with deleterious effects on women’s quality of life in terms of physical, emotional, and social compromise. It is an inflammatory disease characterized by the presence of endometrial-like tissue outside the uterus, and its presentation varies from superficial peritoneal lesions to deep infiltrative endometriosis and ovarian endometrioma. In our previous study, endometriotic lesions were implicated in cellular senescence as their inflammatory pattern could potentially compromise surrounding tissue integrity, thereby inducing a senescent state in cells. P16^Ink4a^ and lamin b1 are biomarkers used to assess cellular senescence. Indirect immunofluorescence staining is a broad technique used to assess cellular structure and behavior driven by protein–protein interactions that provide valuable information about cell functioning. The etiopathogeny of endometriosis is not completely understood and diagnostic approaches still rely on invasive methods; therefore, it is important to use validated methods to increase our understanding of the disease and the development of novel diagnostic tools. However, indirect immunofluorescence protocols are often tissue specific and, if neglected, can lead to misinterpretation of results. Moreover, no valid endometriotic tissue-specific colocalization immunofluorescence protocols have been established. Thus, we have validated a well-funded and suitable protocol to allow precise evaluation of the three presentations of endometriosis lesions using indirect immunofluorescence aiming to support further investigations in endometriosis lesions.

## Introduction

Endometriosis is a gynecological condition characterized by the presence and growth of endometrial-like tissues (both glandular and/or stromal components) outside the uterus^1^. It affects 10% of women in their reproductive period^2–4^. Its main clinical features are pelvic pain and infertility^5^, which can significantly impact women’s quality of life, especially their physical, mental, and sexual life, as well as social well-being and productivity^6–8^. This condition is primarily diagnosed using specific imaging examinations, but some patients have to undergo laparoscopic surgery for histological confirmation of endometriotic lesions^5^.

Endometriotic lesions can be classified into three different types—superficial peritoneal lesions, deep infiltrative endometriosis, and ovarian endometriosis (endometrioma)^9^. Endometriotic tissues are composed of stromal cells with or without glandular cells. Stromal cells present eutopic endometrium-like morphology and usually express estrogen and progesterone receptors on their surface. Moreover, glandular epithelial cells are rarely present separated from the stromal compartment^10,11^. Endometriotic tissue biopsies excised from patients who underwent surgical treatment sometimes can present only with evidence of chronic hemorrhage (hemosiderin laden or foaming macrophages)^12^ once the size of the lesions is poorly correlated to clinical symptoms^13^, ultimately compromising histological diagnosis.

Despite being hormone related, endometriosis is an inflammatory disease^5,14,15^. Its inflammatory pattern is mostly due to the overproduction of cytokines and pro-inflammatory factors, as well as the widespread production of reactive oxygen species (ROS) in response to the presence of ectopic tissue inside the peritoneal cavity^16,17^ and immune response dysregulation, such as overactive macrophages, neutrophils, and natural killer cells^18,19^. Consequently, increased ROS concentration in the pelvic compartment induces oxidative stress^1^, which can induce surrounding tissue damage, leading to a chronic inflammatory pattern associated with adhesion and growth of endometriotic lesions^20^. Finally, the ROS-induced inflammatory microenvironment can impair cellular function, triggering irreversible damages to protein, lipid, and DNA molecules and cell membranes^21^. Cellular impairment can ultimately lead to apoptosis or cell cycle arrest^22^.

In our previous study, higher p16 and depleted lamin b1 concentrations was observed in deep infiltrating endometriosis compared to that in the eutopic endometrium of patients with endometriosis to assess senescence^23^. Senescence is defined as irreversible cell cycle arrest in response to a stimulus, a natural process that occurs in almost every somatic cell in multicellular organisms^24^ and despite their inability to duplicate, senescent cells continue to synthesize metabolites with deleterious effect on surrounding cells and tissues^25,26^. Furthermore, senescence is characterized by changes in chromatin and gene expression, resistance to apoptosis, and acquisition of the senescence-associated secretory phenotype^26^. This milieu induces an inflammatory and oxidative stress state in senescent cells^27^, a feature that is also observed in endometriotic lesions. P16 and lamin b1 are biomarkers currently used to assess cellular senescence^28–30^. Indirect immunofluorescence is a technique used to assess cellular senescence as it provides reliable analysis of colocalized protein expression^31^. However, it is challenging to define a reliable protocol to perform double-target immunofluorescence because it must be replicated and suitable to evaluate a specific tissue of interest.

Therefore, this study aimed to establish a valid and reliable indirect immunofluorescence protocol to evaluate colocalization of senescence biomarkers in the three different presentations of endometriosis (peritoneal, deep infiltrative, and endometrioma) under the assumption of accumulation of inflammatory compounds and widespread oxidative stress in the peritoneal cavity. In addition, endometriotic cellular, epithelial glandular, and non-epithelial compartments were assessed using colocalization staining. This is critical in a disease that remains poorly understood and has diagnostic pitfalls. Despite recent improvements in imaging assessments, the standard diagnostic tool still relies on invasive methods.

## Materials and methods

### Patients and sampling

This study was conducted at Hospital Israelita Albert Einstein (HIAE) as part of the Women’s Health Program of HIAE. This study included patients who had been referred for laparoscopic surgery from September 2019 to June 2021. A single gynecological surgeon (S.P.) categorized patients into two groups according to the presence and absence of endometriosis.

Patients with a proven histological diagnosis of endometriosis were included in the endometriosis group, while those who were not diagnosed with endometriosis were included in the non-endometriosis group. In the endometriosis group, samples of the eutopic endometrium (n = 3) and lesions were collected from patients who had been referred for laparoscopic surgery for the treatment of deep endometriosis (n = 3), superficial endometriosis (n = 3), or ovarian endometrioma (n = 3). In the non-endometriosis group, samples of the endometrium were collected from patients (n = 5) who underwent laparoscopy for the treatment of other diseases such as uterine fibroids and benign ovarian cysts without endometriosis foci, which was confirmed intraoperatively. Written informed consent was obtained from all participants prior to the collection of tissue samples. This study was approved by the Research Ethics Committee of HIAE (CAAE: 56229916.9.0000.0071).

Women aged 18–50 years with eumenorrheic menstrual cycles ranging from 24 to 35 days and without a history of hormonal therapy, including GnRH analog, progestin, and oral hormonal contraceptive therapy, for 6 months prior to surgery were included. Smokers and women with hydrosalpinx and endometrial polyps, diabetes mellitus or any endocrinopathies, cardiovascular disease, dyslipidemia, rheumatological disease such as systemic lupus erythematosus, and oncological diseases were excluded.

### Study design

This study was conducted as a qualitative descriptive study since it constituted a pilot and an explanatory study to enhance awareness on the existing gap in the current literature on colocalization of biomarkers in endometriotic lesions in the three different presentations of endometriosis. A qualitative description is suitable for addressing a straight description of a phenomenon, as no other studies have been proposed before^32^.

### Immunofluorescence

Endometriotic, eutopic, and non-endometriosis tissue samples collected intraoperatively were transported immediately to the experimental research laboratory of HIAE and placed in OCT Tissue Tek (TissueTek^®^ O.C.T, Sakura, Torrance, CA, USA) prior to freezing at − 80 °C, where they were stored until the experiments were performed. Cryostat sections measuring 4 µm from each sample were prepared, including positive control (tissue of endometrial cancer or a random ovary) and negative control (the tissue itself) samples; the latter was not labeled for primary antibody to validate the technique.

To locate target proteins, indirect immunofluorescence was performed using a previously reported protocol^33^ with appropriate modifications for protein colocalization of p16 and lamin b1 with E-cadherin (an epithelial cell marker). Briefly, all samples were thawed, fixed in 4% paraformaldehyde, washed with 0.5% Tween in phosphate-buffered saline (PBS; pH 7.4), and covered with Triton X-100 0.2% solution.

The lamin b1 protocol was established after three tests to determine which of the two available antibodies against lamin b1, rabbit polyclonal immunoglobulin G (H90, catalog no. Sc-20682; Santa Cruz Biotechnology, Dallas, TX, USA) and rabbit monoclonal immunoglobulin G ([EPR8985(B)], ab133741; Abcam, Cambridge, UK), interacted better with the tissue of interest. Subsequently, after defining the lamin b1 antibody (primary antibody 1), another nine dilution assays were performed to establish the interaction between the two primary antibodies, anti-lamin b1 and anti-E-cadherin (primary antibody 2). These assays were conducted with both primary antibodies to determine the dilution that might reduce any potential artifacts in the technique. Prediluted E-cadherin (E-cadherin clone NCH-38 ready-to-use 204, Dako Omnis, Santa Clara, CA, USA) was used as primary antibody 2 in the colocalization immunofluorescence protocol; therefore, the lamin b1 concentration was related to its dilution in prediluted E-cadherin.

Dilutions of 1 µL lamin b1 to 100 µL E-cadherin (1:100) and 2 µL lamin b1 to 100 µL E-cadherin (1:50) were tested. For secondary antibodies, to determine which type of fluorescein better interacted with the primary antibodies, four different fluorescein antibodies were tested—Alexa Fluor^®^ 488 (Goat Anti-197 Rabbit IgG H&L [ab150077], Abcam, Cambridge, UK) and Alexa Fluor^®^ 680 (goat anti-rabbit IgG H&L [ab175773], Abcam, Cambridge, UK) for primary antibody 1 and conventional fluorescein (FITC—goat anti-mouse polyclonal antibody, STEMCELL Technologies, Vancouver, BC, Canada) and Alexa Fluor^®^ 647 (goat anti-mouse IgG H&L pre 198 adsorbed [ab150115]) for primary antibody 2. Secondary antibodies were tested following dilutions of 1:200 and 1:400. The incubation period of primary antibodies (E-cadherin and lamin b1) was assessed after three tests of 16, 18, and 24 h as non-specific staining results were an issue during the first tests. Finally, to avoid excessive primary and secondary antibody labeling around the tissue, wash tests were performed, in which the washing solution was instantly or placed for 5-min before it was removed.

The p16^Ink4a^ immunofluorescence protocol was established after three dilution tests, in which 1 µL of p16^Ink4a^ to 100 µL of E-cadherin (1:100), 0.5 µL of p16^Ink4a^ to 100 µL of E-cadherin (1:200), 0.67 µL of p16^Ink4a^ to 100 µL of E-cadherin (1:150), and 2 µL of p16^Ink4a^ to 100 µL of E-cadherin (1:50) were tested. Like both lamin b1 and p16^Ink4a^ (rabbit monoclonal 200 [EPR1473] to CDKN2A/p16INK4a tag; Abcam, Cambridge, UK) used had the same isotype (rabbit antibodies), the same stated for lamin b1 concerning secondary antibodies were done to p16^Ink4a^ as well as the attempt to decrease overlabeling.

Photomicrography was performed using a Carl Zeiss LSM 710 confocal microscope (Oberkochen, Germany) and Zen 2010 software (blue edition; version 6.0; Zeiss; Germany) for image analysis. Lamin b1-, p16-, and E-cadherin-stained endometrial tissue slides were visualized using pre-settings following a limited range for all images. First, the channels of continuous-wave laser were set to achieve the spectrum of the secondary antibody emission profile, which had to include three different channels to detect 4′,6-diamidino-2-phenylindole (DAPI; detection wavelength: 410–507 nm), E-cadherin secondary antibody (FITC or Alexa Fluor^®^ 647), and lamin b1 or p16 secondary antibodies (Alexa Fluor^®^ 488 or Alexa Fluor^®^ 680). The colors for each channel were also pre-set; green, red, and blue were chosen for E-cadherin (Alexa Fluor^®^ 488 or Alexa Fluor^®^ 680), lamin b1 or p16^Ink4a^ (FITC or Alexa Fluor^®^ 647), and DAPI, respectively.

Subsequently, slides of the tissue of interest were placed on the confocal microscopy compartment, and then, the negative control was firstly detected using microscopy (20× magnification), to determine acquisition settings. Live images were displaced to manually configure the following: laser power, which was individually set to each slide; pinhole size for acquisition, which was set preferentially at 1 AU (38 microns); digital gain; and digital offset. These settings were designed to achieve the lowest laser power and gain in the system to obtain images without saturating the pixels. For all acquisitions, images were integrated with a 6.30-µs pixel time, 30.98-s frame time, and 30-µs line time. The image size was scaled at 425.10 µm × 425.10 µm. The signals are integrated into 12 bits. Images were taken at 20× and both 63× and 100× immersion oil magnifications. Images were viewed and modified using Pixlr © (Inmagine Lab Pte Ltd 2021). The schematic slide model was developed using Adobe Illustrator (version CC 2019; Copyright © 2021 Adobe).

### Ethics approval

Ethical approval was waived by the Research Ethics Committee of Hospital Israelita Albert Einstein (protocol code CAAE: 56229916.9.0000.0071 and date of approval 09/06/2017).

### Consent to participate

Informed consent was obtained from all subjects involved in this study.

## Results

**Figure 1:**
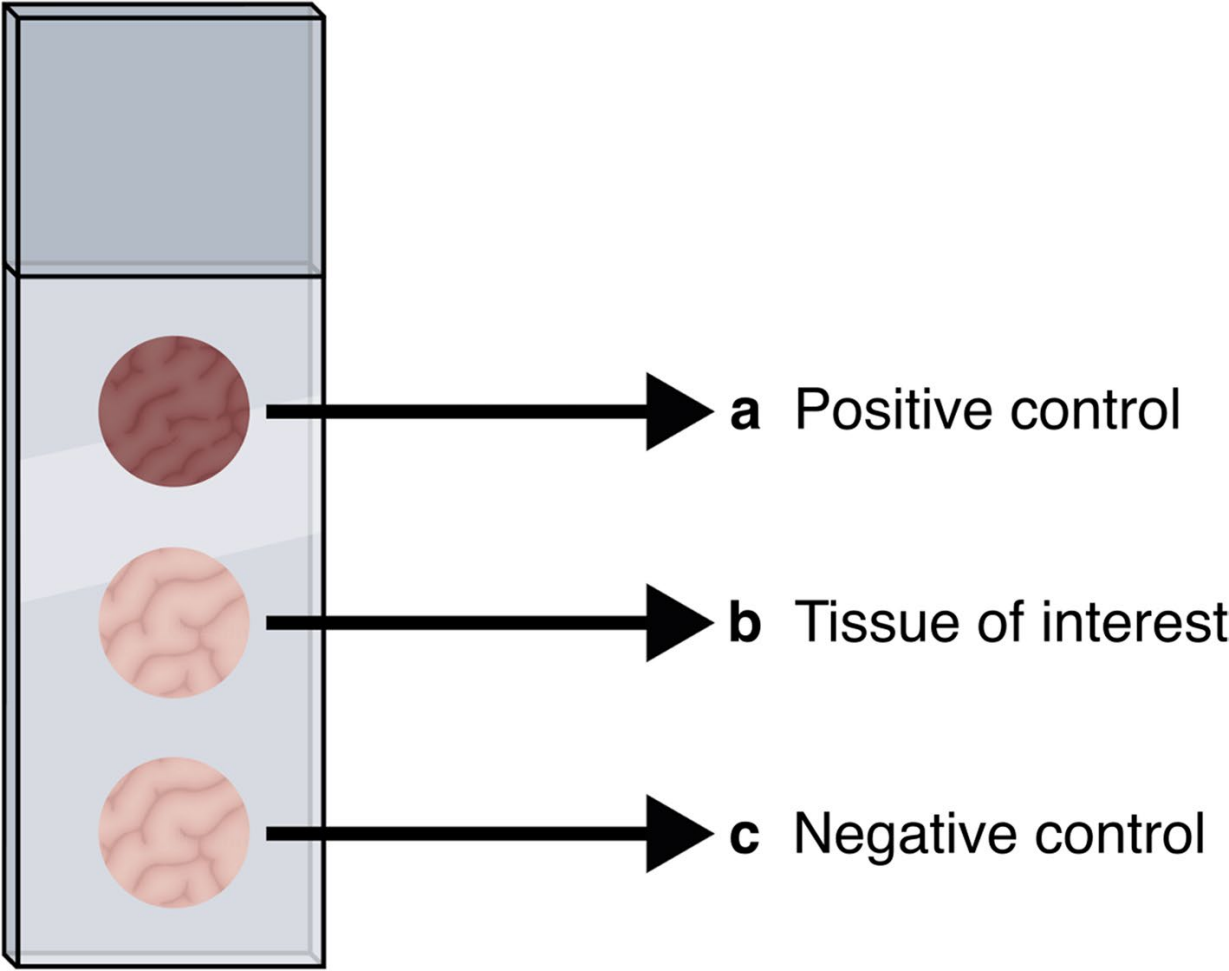
Standardized structure scheme of the slides for the immunofluorescence staining. (**a**) Positive control—represented by samples of a random ovary or an endometrial cancer—was assessed to strengthen the technique to eliminate any mislabeling (**b**) samples of the interest tissues—peritoneal, deep infiltrating or ovary endometriosis and the eutopic or non-endometriosis endometrium (**c**) an additional sample of the interest tissue itself represented the negative control to mitigate any labeling mistake of the antibodies.

The immunofluorescence protocol for colocalization of senescence-related proteins in endometriotic lesions followed an approach to refine the labeling technique and reduce any potential artifacts. Since there is a gap in the literature regarding colocalization in endometriotic tissues, we assumed that the tests should be backed up by a non-endometrial-like tissue positive control and a negative control to strengthen the protocol. The slides were structured as shown in Fig. 1.

### p16 incubation test

Briefly, slides were firstly thawed, fixed, and washed, as described in the Methods section. Then, the primary antibody solution of p16^Ink4a^ and prediluted monoclonal anti-E-cadherin was prepared at proper concentrations—1 µL of p16^Ink4a^ in 100 µL of prediluted E-cadherin (1:100)—and placed in each section for overnight incubation, except the negative control section, which was incubated in PBS at 4 °C for 24 h. The slides were then washed with 0.5% Tween in PBS. FITC and Alexa Fluor^®^ 680 were diluted in 0.5% Tween in 5% bovine serum albumin solution (BSA)—(1:400)—and then placed over the samples for 1-h incubation at 23–26 °C. Finally, the slides were covered with DAPI to stain the cell nuclei.

In an additional assay, p16 was incubated at a concentration of 1:100 with prediluted E-cadherin. However, secondary antibodies were changed. Alexa Fluor^®^ 488 and Alexa Fluor^®^ 647 were used to bind primary antibodies 1 and 2, respectively. They were assessed at a 1:400 dilution. The incubation period did not exceed 16 h. To achieve a better standard for the captured images, another incubation test was performed. Briefly, there was no change in p16 diluted in E-cadherin and secondary antibodies; however, the incubation period was changed to 18 h. After overnight incubation, the samples were covered with 0.5% Tween and washed with PBS three times for 5 min. This process was repeated after incubation with the secondary antibodies.

### Lamin b1 incubation test

The same procedure as that used for p16 in the first steps of indirect immunofluorescence was performed for lamin b1. Unlike p16, lamin b1 showed huge labeling differences between the two tested anti-lamin b1 antibodies from different laboratories for both endometriotic and positive control tissues. Rabbit monoclonal antibody to lamin b1 (tag: abkar) and rabbit polyclonal antibody to lamin b1 (tag: Santa Cruz Biotechnology) were tested individually and in prediluted E-cadherin, both at a dilution of 1:100. The incubation period was 24 h. The secondary antibodies FITC (1:100) and Alexa Fluor^®^ 680 (1:100) were diluted in 0.5% Tween with 5% BSA solution and incubated for 1 h. Similar to the procedure for p16, slides were covered with DAPI. A reliable target pattern was achieved by the rabbit polyclonal antibody to lamin b1 (tag: Santa Cruz Biotechnology).

Furthermore, polyclonal lamin b1 antibody dilutions of 1:100 and 1:50 were used, the secondary antibodies were changed, Alexa Fluor^®^ 488 and Alexa Fluor^®^ 647 were used at dilutions of 1:400, and the tissues were incubated for 18 h. Furthermore, lamin b1 labeling at a concentration of 1:50 and 24 h incubation, together with 3 washes with 0.5% Tween with PBS lasting 5 min, was then assessed.

**Figure 2:**
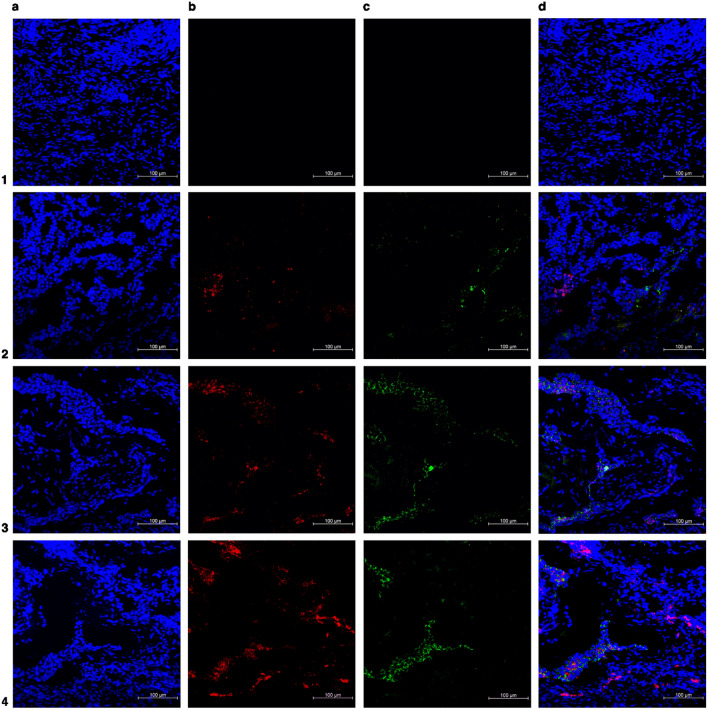
Representative immunofluorescence costained images of ovarian endometrioma. (**a**) In blue: DAPI (**b**) in red: senescent associated biomarkers (p16 or lamin b1) (**c**) in green: anti-E-cadherin antibody (epithelial glandular cells) (**d**) merged. Number 1 row represents negative control (endometrioma), number 2 row represents positive control (endometrial cancer or random ovary), number 3 row represents endometrioma labeled with p16, and number 4 row represents endometrioma labeled with lamin b1. ×20 magnification.

**Figure 3:**
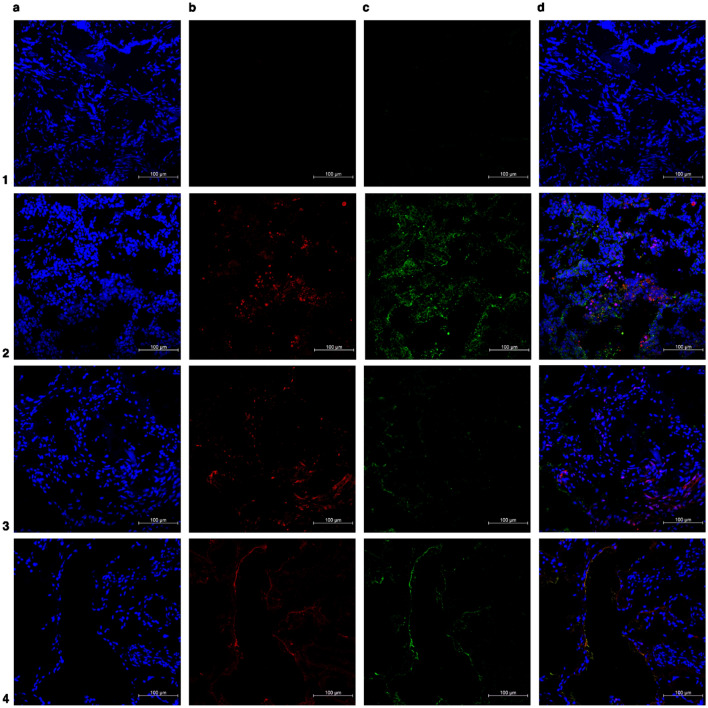
Representative immunofluorescence costained images of superficial peritoneal endometriosis. (**a**) In blue: DAPI (**b**) in red: senescent associated biomarkers (p16 or lamin b1) (**c**) in green: anti-E-cadherin antibody (epithelial glandular cells) (**d**) merged. Number 1 row represents negative control (endometriotic superficial lesions), number 2 row represents positive control (endometrial cancer or random ovary), number 3 row represents superficial endometriosis labeled with p16, and number 4 row represents superficial endometriosis labeled with lamin b1. ×20 magnification.

**Figure 4:**
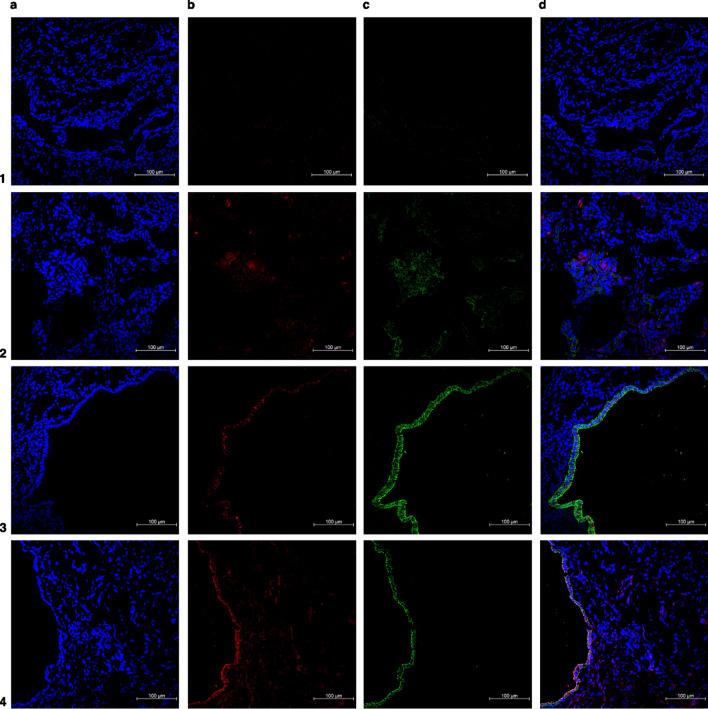
Representative immunofluorescence co-stained images of deep infiltrating endometriosis. (**a**) In blue: DAPI (**b**) in red: senescent associated biomarkers (p16 or lamin b1) (**c**) in green: anti-E-cadherin antibody (epithelial glandular cells) (**d**) merged. Number 1 row represents negative control (deep infiltrative endometriotic lesion), number 2 row represents positive control (endometrial cancer or random ovary), number 3 row represents deep infiltrating lesion labeled with p16, and number 4 row represents deep infiltrating lesion labeled with lamin b1. ×20 magnification.

**Figure 5:**
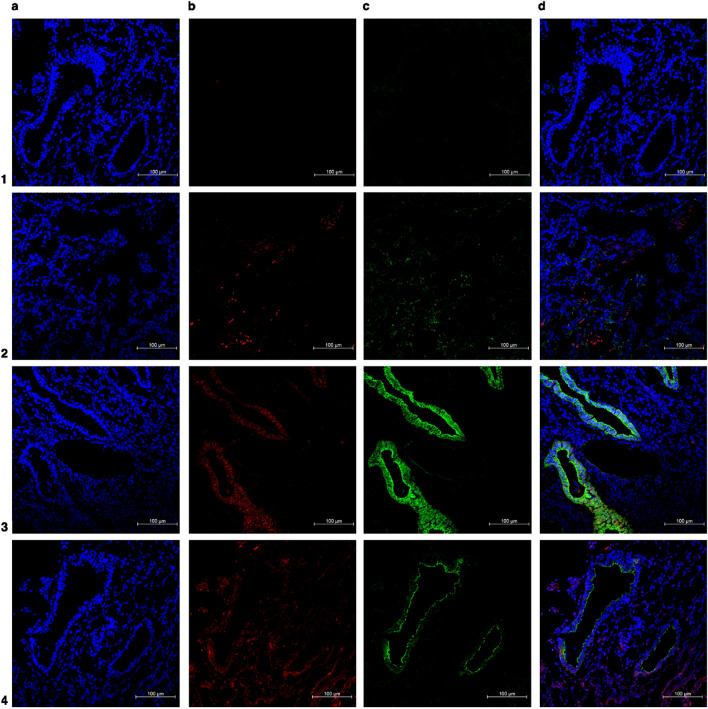
Representative immunofluorescence costained images of the eutopic endometrium in the endometriosis group. (**a**) In blue: DAPI (**b**) in red: senescent associated biomarkers (p16 or lamin b1) (**c**) in green: anti-E-cadherin antibody (epithelial glandular cells) (**d**) merged. Number 1 row represents negative control (eutopic endometrium of endometriosis group), number 2 row represents positive control (endometrial cancer or random ovary), number 3 row represents the eutopic endometrium labeled with p16, and number 4 row represents the eutopic endometrium labeled with lamin b1. ×20 magnification.

**Figure 6:**
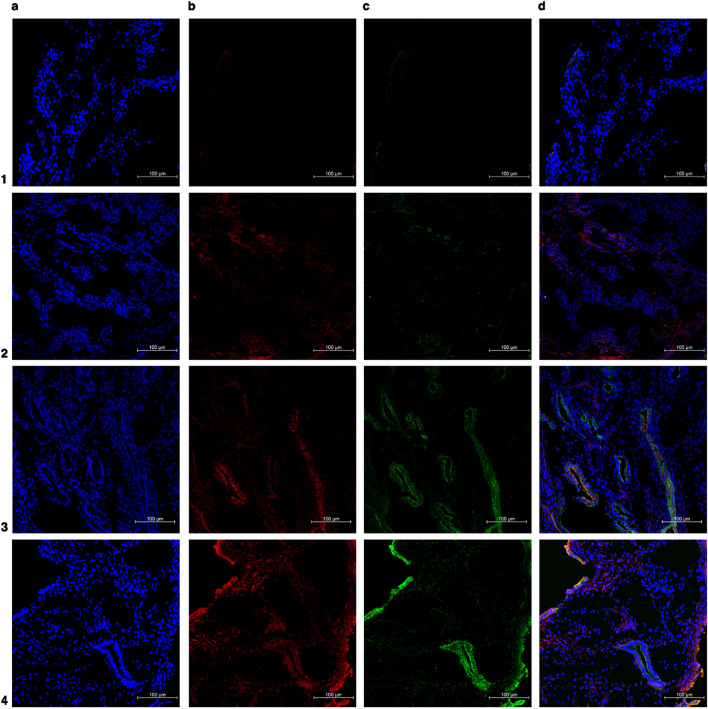
Representative immunofluorescence costained images of the endometrium in the non-endometriosis endometrium. (**a**) In blue: DAPI (**b**) in red: senescent associated biomarkers (p16 or lamin b1) (**c**) in green: anti-E-cadherin antibody (epithelial glandular cells). (**d**) merged. Number 1 row represents negative control (endometrium of control group), number 2 row represents positive control (endometrial cancer or random ovary), number 3 row represents non-endometriosis endometrium labeled with p16, and number 4 row represents control group endometrium labeled with lamin b1. ×20 magnification.

Based on these results, the protocol enlisted in Tables 1 and 2 was established. This protocol outlines the basic steps for either direct or indirect costaining immunofluorescence labeling of the three different presentations of endometriotic lesions. Slides were analyzed using a Carl Zeiss LSM 710 confocal microscope (Oberkochen, Germany) fitted with Zen 2010 software for image analysis. Analysis was performed with endometrioma (Fig. 2), peritoneal endometriosis (Fig. 3), deep infiltrative endometriosis (Fig. 4), the eutopic endometrium (Fig. 5), and the non-endometriosis endometrium (Fig. 6).

**Table 1:** p16 immunofluorescence staining protocol for endometriosis tissue.

**Day 1**	
1	Cryostat 4-µm thick sections previously sliced on charged slides and stored at − 80 °C should be collected and dried at room temperature (23–26 °C)
2	Draw a circle around each tissue section using hydrophobic barrier pen to keep reagents localized on tissue specimens, avoiding waste of materials, and preventing mix of reagents
3	Cover the tissues sections with 4% paraformaldehyde solution and incubate for 15 min for tissue fixation
4	Wash sections three times with 0.5% Tween solution—no need for time incubation at this wash step
5	Incubate sections with Triton-X 100 0,2% solution for 15 min
6	Wash sections for three times with 0.5% Tween solution—no need for time incubation at this wash step
7	Block the tissue covering the slides with 5% BSA solution for 30 min
8	Wash sections for three times with 0.5% Tween solution—no need for time incubation at this wash step
9	Prepare the primary antibodies solution following the dilution displayed below:
**Dilution of p16 (1:100) in prediluted E-cadherin**	
1 µL of p16 in 100 µL of prediluted E-cadherin	
Note: If the second biomarker chosen for colocalized immunofluorescence is not prediluted, a 0.5% Tween and 5% BSA solution should be used to meet the required dilution for both primary antibodies	
10	Cover the tissue sections of the positive control and the interest tissue with the primary antibody solution, while the negative control should be covered with PBS
11	Gently put the slides in a humidified chamber to overnight incubation (18 h) at 4 °C
**Day 2**	
1	Take the chamber containing the slides from the freezer and gently place it at room temperature (23–26 °C)
2	Wash sections for three times with 0.5% Tween solution for 5 min—this step is particularly important to avoid overlabeling by the primary antibodies
3	Prepare the secondary antibodies solution together with 0.5% Tween and 5% BSA solution following the dilution displayed below:
**Dilution secondary antibodies (Alexa Fluor**^**®**^** 488 and Alexa Fluor**^**®**^** 647)—1:400**	
1 µL of Alexa Fluor® 488 + 1 µL Alexa Fluor® 647 in 400 µL of 0.5% Tween and 5% BSA solution	
4	Cover all the tissue sections with the secondary antibody solution for 1 h at room temperature. If available, chose a dark chamber to rest the slides during this incubation period to avoid photobleaching
5	Wash sections for three times with 0.5% Tween solution for 5 min—this step is particularly important to avoid overlabeling by the secondary antibodies
6	Let the slides dry at room temperature
7	Stain nuclei with DAPI and mount with mounting medium

**Table 2:** Lamin b1 immunofluorescence staining protocol for endometriosis tissue.

**Day 1**	
1	Cryostat 4-µm thick sections previously sliced on charged slides and stored at − 80 °C should be collected and let dry at room temperature (23–26 °C)
2	Draw a circle around each tissue section using hydrophobic barrier pen to keep reagents localized on tissue specimens—avoiding waste of materials and preventing mix of reagents
3	Cover the tissues sections with a 4% paraformaldehyde solution and let them incubate for 15 m for tissue fixation
4	Wash sections three times with 0.5% Tween solution—no need for time incubation at this wash step
5	Incubate sections with a 0.2% Triton-X 100 solution for 15 min
6	Wash sections three times with 0.5% Tween solution—no need for time incubation at this wash step
7	Block the tissue covering the slides with a 5% BSA solution for 30 min
8	Wash sections three times with 0.5% Tween solution—no need for time incubation at this wash step
9	Prepare the primary antibodies solution following the dilution displayed below:
**Dilution of lamin b1 (1:50) in prediluted E-cadherin**	
2 µL of lamin b1 in 100 µL of prediluted E-cadherin	
Note: If the second biomarker chosen for colocalized immunofluorescence is not prediluted, a 0.5% Tween and 5% BSA solution should be used to meet the required dilution for both primary antibodies	
10	Cover the tissue sections of the positive control and the interest tissue with the primary antibody solution, while the negative control should be covered with PBS
11	Gently put the slides in a humidified chamber to overnight incubation of 24 h in 4 °C
**Day 2**	
1	Take the chamber containing the slides from the freezer and gently place it at room temperature (23-26 °C)
2	Wash sections for three times with 0.5% Tween solution for 5 min—this step is particularly important to avoid overlabeling by the primary antibodies
3	Prepare the secondary antibodies solution together with 0.5% Tween and 5% BSA solution following the dilution displayed below:
**Dilution secondary antibodies (Alexa Fluor**^**®**^** 488 and Alexa Fluor**^**®**^** 647)—1:400**	
1 µL of Alexa Fluor® 488 + 1 µL Alexa Fluor® 647 in 400 µL of 0.5% Tween and 5% BSA solution	
4	Cover all the tissue sections with the secondary antibody solution for 1 h at room temperature. If available, chose a dark chamber to rest the slides during this incubation period to avoid photobleaching
5	Wash sections for three times with 0.5% Tween solution for 5 min—this step is particularly important to avoid overlabeling by the secondary antibodies
6	Let the slides dry at room temperature
7	Stain nuclei with DAPI and mount with mounting medium

Moreover, immunofluorescence staining is far more complex than a single-approach protocol because the technique can change with antibody and tissue selection for specific applications. Therefore, Tables 1 and 2 and Figs. 2, 3, 4, 5, 6 summarize our data on this technique for the three different presentations of endometriosis that we consider the most reproducible and reliable, which do not spare a judicious evaluation of the protocol and materials that will be employed, particularly antibody selection; therefore, adjustments are fairly encouraging. Our main goal was to ensure that some nuances that are often neglected (incubation period, wash pattern, and differences between antibodies) were highlighted to further improve endometriotic lesion assessments.

All methods were carried out in accordance with relevant guidelines and regulations.

## Discussion

Endometriotic lesions can present as different morphological patterns, varying from superficial peritoneal lesions to nodules exceeding 5 mm in penetration depth (deep endometriosis) and ovarian cysts (endometrioma)^34^. However, it is unclear whether this plethora represents different presentations of the same disease or distinct entities.

Despite its benign pattern, this condition has several implications on women’s quality of life, with debilitating symptoms that overlap with other inflammatory gynecological conditions and therefore diagnosis is mostly delayed^35^. Non-invasive methods to diagnose endometriosis rely on image assessments that are costly and demand highly trained personal. Reliable diagnostic biomarkers could potentially overcome this diagnostic gap in endometriosis but so far remain elusive^36^.

To completely understand the machinery of any given cell or tissue and provide accurate molecular assessments, it is pivotal to identify the framework behind its morphology and interactions between its molecules and their compartments^37^. Knowledge of different methods that allow precise and robust evaluation of endometriotic lesions is crucial to address the existing gap on the presentation and etiology of endometriosis and further enhance molecular assessment of endometriotic lesions. Immunofluorescence is a recognizable approach driven by protein–protein interactions that provides valuable findings on cellular functioning through visual interpretation and quantitative analysis^38^.

Our previous studies revealed a decrease in lamin B1 expression^28^ and an increased expression of p16^23^ in deep infiltrative endometriosis lesions compared to that in the eutopic endometrium, which could potentially be implicated in the pro-senescence pattern of endometriosis lesions as both lamin b1 and p16 are biomarkers used to assess cellular senescence. However, this study is anyhow intended to highlight this assumption as this aim has been detailed elsewhere. In this study, we focused on standardizing an indirect colocalization immunofluorescence protocol suitable for endometriotic lesions.

**Figure 7:**
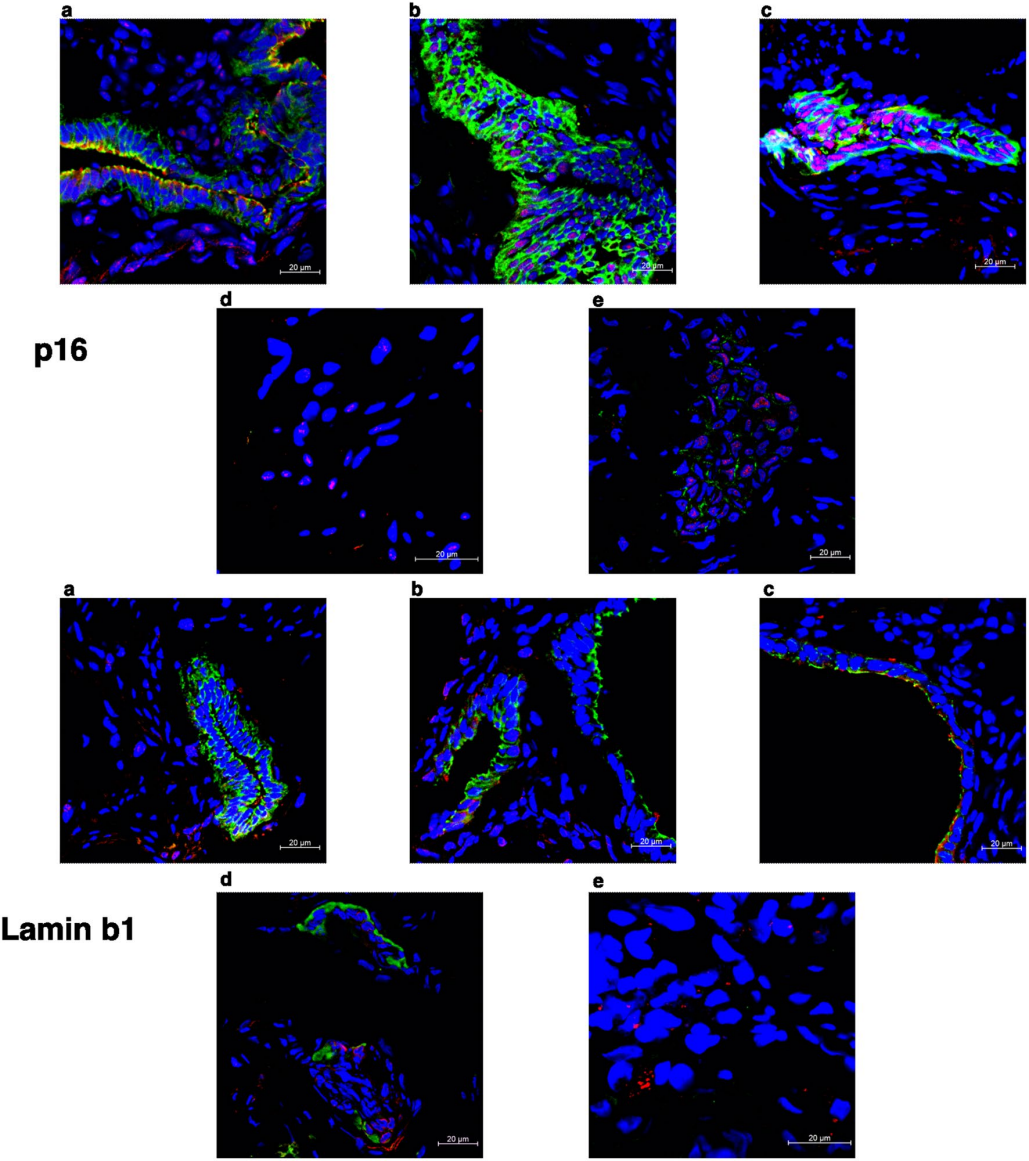
Representative images of endometriosis and non-endometriosis tissues costained with p16^Ink4a^ or lamin b1 and E-cadherin (epithelial cell marker). p16^Ink4a^ and lamin b1 are shown in red, E-cadherin is shown in green, and the cell nuclei is shown in blue (DAPI). (**a**) Control endometrium, (**b**) eutopic endometrium, (**c**) deep endometriosis lesion, (**d**) superficial endometriosis lesion, (**e**) endometrioma. Pictures were acquired at ×63 or ×100 oil magnification.

In our study, high-quality images showed lamin b1 and p16 labeling patterns on the three distinct endometriotic lesions, eutopic endometrium, and non-endometriosis endometrium along with their glandular-epithelial and non-glandular-epithelial compartments (Figs. 2, 3, 4, 5, 6). p16 labeling seemed colocalize with E-cadherin (glandular-epithelial cell marker), which was overexpressed in the epithelial compartment in samples from the non-endometriosis endometrium (Fig. 7a), eutopic endometrium (Fig. 7b), and deep infiltrative endometriosis groups (Fig. 7c). Furthermore, in these samples, even in the non-epithelial compartment, p16 showed discrete labeling. Superficial peritoneal (Fig. 7d) and ovarian endometrioma (Fig. 7e) samples were not labeled with E-cadherin, and in the non-epithelial compartment, p16 was underlabeled.

Lamin b1 staining seemed to have an increased labeling distribution in the non-endometriosis endometrium (Fig. 7a), eutopic endometrium (Fig. 7b), and deep infiltrative endometriosis (Fig. 7c) groups, not only in the non-epithelial compartment but also when co-localized with E-cadherin, indicating the presence of significant glandular components in these samples. Superficial peritoneal (Fig. 7d) and ovarian endometrioma (Fig. 7e) samples, as shown by p16 staining, had no expressive epithelial component once they were poorly labeled with E-cadherin; however, when present epithelial component, lamin b1 labeling was also observed.

Immunofluorescence staining is a widely used technique that allows visualization of many components and cell compartments in any tissue or cell type using a combination of a specific antibody and fluorophore^39^. Despite its wide applicability, this labeling technique is not easy as it is very specific to the types of tissue and antibodies^1^. Therefore, optimal materials and methods should be determined empirically based on the sample and antigen type. However, a reliable protocol for a particular tissue or cell type of interest may contribute to shortening the empirical phase, despite the fact that dilution and incubation assays should be performed to assess the properties of antibodies of interest.

To ensure successful immunofluorescence, critical parameters must be met. Fixation is the first crucial step to assure cellular component integrity while allowing immobilization of target antigens and further maximizing antibody-epitope interaction^31^. Permeabilization is the second approach to optimize epitope exposure by denaturation of fixed proteins^40^. Both conditions must be assessed considering not only the tissue or cell type but also the antibody itself. The protocol stated in this study was outlined to address the interaction of the nuclear envelope (p16 and lamin b1) and intercellular junction (e-cadherin) antibodies. Paraformaldehyde, an organic solvent, was chosen as the fixative agent and triton-100 X as the permeabilization agent. Most studies using immunofluorescence alone or with techniques in their methodology for endometriosis have rarely provided a summary of the procedure itself, and they often did not reference the protocol in their analysis, which could have the potential to compromise the study integrity once the sensitivity for mislabeling in this technique is well known^41^.

Background staining with secondary antibodies is occasionally too high; therefore, sorbing reagents can be used to mitigate this phenomenon. BSA effectively blocks nonspecific sites. The rationale behind using a different specie of serum-based blocking buffer is that it would prevent cross-reactivity with host protein antibodies, which would probably yield low background staining^42^.

Following these steps, direct or indirect immunofluorescence assays could be performed. Although the direct approach is useful and quicker, the indirect method is used because it has higher sensitivity, and different antibodies and different targets can be concurrently on a sample^43^, which was the aim of this study. Both techniques can be performed by following the protocol outlined above, whereas the second incubation step is only required for the indirect method. After the preliminary phase of fixation, permeabilization, and tissue blocking, a two-step incubation process is performed.

First, the primary antibodies of interest are incubated to bind the target epitopes. At this point, it is imperative to perform the incubation period assays to elucidate the optimal time of incubation for labelling the tissue samples. In our study, lamin b1 required a longer incubation period than p16 (24 h vs. 18 h) to achieve a satisfactory staining quality. Individual tests should be performed before using multiple target antigens. Primary antibodies must be from a different species chosen species and the secondary antibodies must be the same as the chosen species. This precaution is particularly important to avoid cross-reactions between tissue samples of endogenous immunoglobulins and the primary antibody^44^. For this immunofluorescence protocol, we used lamin b1 and p16, rabbit polyclonal and monoclonal antibodies, respectively, and its corresponding anti-rabbit secondary antibody, Alexa Fluor^®^ 488 goat anti-197 rabbit IgG. E-cadherin was selected from a different species from lamin b1 and p16, a mouse anti-human antibody, and Alexa Fluor^®^ 647 goat anti-mouse IgG was used as the secondary antibody. Therefore, the next and final step is the incubation of the corresponding secondary antibodies to bind with the primary antibodies for 1 h at room temperature, staining the nuclei with DAPI and mounting the slides using mounting media. Most endometriosis studies applying immunofluorescence do not follow a well-structured protocol; for instance, the incubation temperature is not always evident and incubation at a specific temperature is often neglected, which can compromise the staining as optimal temperatures are required to allow the epitope–protein interaction to improve fluorescent immunolabeling, possibly through increased penetration^45^ of the antibody.

We would also strongly recommend that a trained pathologist should be consulted to review the staining patterns for each antibody to assess its appropriate localization in the cell (membrane and cell nuclei) and evaluate its co-localization with other markers used.

Washing after antibody incubation is an important step^46^. As mentioned earlier, background staining can be tricky, thus allowing the wash solution to incubate for a short period of time can prevent this issue. This protocol allows washing the slides sections three times after exposure to both primary and secondary antibodies and lets the detergent solution act for 5 min before the next wash. Most immunofluorescence studies on endometriosis do not indicate these nuances.

Another particularity regarding nonspecific staining and overtargeting of the secondary antibody that must be considered to ensure consistent performance and reproducibility of staining is the use of positive and negative controls. Sections from a different tissue than the one of interest that expresses the protein of interest should be assessed to allow validity of the staining results as a positive result will indicate whether the immunofluorescence is performed properly. Furthermore, a negative control must also be used; however, certain tissue types can have fluorescent molecules, resulting in false-positive staining and affecting the interpretation of the results^47^. To resolve this issue, a lack of signal needs to be confirmed in a sample incubated only with the secondary antibody, while incubation with the primary antibody should be replaced with a buffer solution. For this study, we developed a structured slide model to assess not only the tissue itself but also the negative and positive controls for each sample to systematically perform the immunofluorescence technique and further analyze the image with certainty. Figure 1 shows the schematic of the slide. The negative control was not incubated with the primary antibody, but it was incubated with PBS, whereas the positive control and the tissue of interest followed the two-step immunofluorescence technique.

As with any other protocols, this protocol outlined has limitations. The staining patterns in our samples were interpreted visually and, although we based our interpretation on previous studies, we acknowledge the fact that visual interpretation is subjective and can be misinterpreted^48^. Subjective visual analysis is highly susceptible to bias; however, we did not consider this limitation as an issue for the strength of our protocol because we wanted to emphasize the particularities of the immunofluorescence technique and establishment of a protocol that was reproducible on the three different presentations of endometriosis. Immunofluorescence is a widely used technique but not easy to execute. A reliable protocol suits well to mitigate longer and stressful empirical phases that could compromise the results. We met these criteria.

Endometriosis is a very common and poorly understood disease, with serious implications on the health of women. Knowledge of cellular behavior is crucial to provide advances in the management of its burden clinical presentation, and reliable assessment methods such as immunofluorescence costaining is essential to guide further analysis. Moreover, non-invasive diagnostic tools could be evolved by accessing biomarkers through this technique - a promising path to uncover unmet diagnostic options.

## Data Availability

The corresponding author on behalf of all authors confirm that the data supporting the findings on this study are available within the article. Raw data that support the findings of this study are available upon reasonable request from the corresponding author.
